# Chestnut Honey Is Effective against Mixed Biofilms at Different Stages of Maturity

**DOI:** 10.3390/antibiotics13030255

**Published:** 2024-03-13

**Authors:** Regina Koloh, Viktória L. Balázs, Lilla Nagy-Radványi, Béla Kocsis, Erika Beáta Kerekes, Marianna Kocsis, Ágnes Farkas

**Affiliations:** 1 Department of Pharmacognosy, Faculty of Pharmacy, University of Pécs, 7624 Pécs, Hungary; kolohregina@gmail.com (R.K.); viktoria.balazs@aok.pte.hu (V.L.B.); lilla.radvanyi@aok.pte.hu (L.N.-R.); 2 Department of Medical Microbiology and Immunology, Medical School, University of Pécs, 7624 Pécs, Hungary; kocsis.bela@pte.hu; 3 Department of Microbiology, Faculty of Science and Informatics, University of Szeged, 6726 Szeged, Hungary; kerekeserika88@gmail.com; 4 Department of Agricultural Biology, Institute of Biology, University of Pécs, 7624 Pécs, Hungary; kocsis.marianna@pte.hu

**Keywords:** chronic wound, MRSA, *Pseudomonas*, *Staphylococcus*

## Abstract

The irresponsible overuse of antibiotics has increased the occurrence of resistant bacterial strains, which represents one of the biggest patient safety risks today. Due to antibiotic resistance and biofilm formation in bacteria, it is becoming increasingly difficult to suppress the bacterial strains responsible for various chronic infections. Honey was proven to inhibit bacterial growth and biofilm development, offering an alternative solution in the treatment of resistant infections and chronic wounds. Our studies included chestnut honey, valued for its high antibacterial activity, and the bacteria *Pseudomonas aeruginosa*, methicillin-resistant *Staphylococcus aureus*, and *S. epidermidis*, known to form multi-species biofilm communities. Minimum inhibitory concentrations (MIC) of chestnut honey were determined for each bacterial strain. Afterwards, the mixed bacterial biofilms were treated with chestnut honey at different stages of maturity (incubation times: 2, 4, 6, 12, 24 h). The extent of biofilm inhibition was measured with a crystal violet assay and demonstrated by scanning electron microscopy (SEM). As the incubation time increased and the biofilm became more mature, inhibition rates decreased gradually. The most sensitive biofilm was the combination MRSA-*S. epidermidis*, with a 93.5% inhibition rate after 2 h of incubation. Our results revealed that chestnut honey is suitable for suppressing the initial and moderately mature stages of mixed biofilms.

## 1. Introduction

Today, one of the biggest patient safety risks is the uninformed and irresponsible overuse of antibiotics, which has led to the emergence of antibiotic resistance. Antibiotic resistance is the inherited ability of microorganisms that allows them to grow at high antibiotic concentrations [1]. Resistant bacteria are able to grow and divide at antibiotic concentrations that kill or stop the growth of other strains of the same species. Bacteria with a biofilm-forming ability are particularly prone to developing resistance against certain antibiotics [2,3]. Bacterial biofilms are complex, surface-attached bacterial communities held together by a self-produced matrix of adherent extracellular polymeric substances (EPS) [4], secreted proteins, and extracellular DNA. In addition to the protection provided by the matrix, bacteria in biofilms can employ a number of survival strategies to avoid the host’s immune response or possibly drug therapy. Within the biofilm, the bacteria adapt to the lack of oxygen in the environment and the limited amount of nutrients by changing the metabolism of the bacteria located in the matrix, as they reduce gene expression and protein production, which leads to a lower rate of metabolism and cell division. Furthermore, these adaptations make bacteria more resistant to antimicrobial therapy. In a biofilm, the antibiotic-bacteria contact is highly influenced. The matrix serves as a physical barrier that is difficult to penetrate [5]. Thanks to lower concentrations of the antibiotic, the bacteria gain time to develop tolerance. The biofilm matrix enables the close proximity of bacterial communities [6] and provides an ideal habitat for the cell exchange of plasmids encoding resistance to antibiotics, thereby potentially promoting the spread of antibiotic resistance [7]. Based on research results, the horizontal gene transfer of resistance genes between bacterial cells within biofilm is 700 times more efficient than between free-living, planktonic bacterial cells [8].

Frequently, bacterial biofilms can be observed as a combined mixture, which in many cases represents an even greater patient safety risk. It is also common on the surface of wounds that different strains create a unit embedded in a common biofilm, which makes treatment even more difficult [3,9]. Chronic wounds are associated with a lack of epithelium and tissue. They are also characterized by the fact that the four phases of wound healing (hemostasis, inflammation, sprouting, and desquamation) are interrupted, and the wound does not heal even within 10–12 weeks. Since the surface of chronic wounds is most often colonized by mixed biofilms, our research was carried out with pathogens that play an important role in the development of chronic wounds and, at the same time, are inclined to form mixed biofilms, such as *Pseudomonas aeruginosa*, methicillin-resistant *Staphylococcus aureus* (MRSA), or *Staphylococcus epidermidis* [10]. *Pseudomonas aeruginosa* is a Gram-negative, opportunistic nosocomial pathogen. The bacterium typically enters the body through carrier objects, catheters, breathing tubes, contaminated drinking water, or food, where it causes serious infections in weakened, immunocompromised, or chronically ill individuals [11]. MRSA is a Gram-positive bacterium that produces several toxins and enzymes that are responsible for its pathogenicity. Cytotoxins cause pore formation and induce inflammation in mammalian cells, which can contribute to sepsis. In the case of injuries, surgical interventions, and weakened immune systems, they can cause local skin infections and other infections. The infection is spread by skin-to-skin contact or by means of equipment that has been in contact with the infected skin surface [12]. *Staphylococcus epidermidis* is also a Gram-positive, opportunistic pathogen that can cause nosocomial infections in immunocompromised patients and neonates, mostly via medical devices, including catheters and other surgical implants and prostheses [13]. Mixed biofilms formed on the surface of wounds by the bacteria mentioned above are extremely resistant to antibiotic therapy [14].

To overcome this, research into new, effective alternative therapies is needed. Wound healing is a rather complex process. Many different methods have been used to treat acute and chronic wounds. However, antibiotic resistance has rendered many antimicrobial agents ineffective in wound management. As a result, alternative remedies have become increasingly popular, one of which is the use of honey as a wound healing agent. Honey effectively inhibits the growth of bacteria and can accelerate the healing of wounds such as burns, scratches, diabetes-related skin abscesses, malignant tumors, leprosy, fistulas, leg ulcers, traumatic wounds, amputations, septic, and surgical wounds. The reason for this is that it contains various biologically active compounds, including flavonoids, phenolic acids, organic acids, enzymes, and vitamins, which help the wound healing process [15].

Depending on the composition of biologically active compounds, the antibacterial efficacy of different varietal honeys can differ to a large extent. Among unifloral honeys, which are derived predominantly from a certain plant species or genus, the amber to dark brown, somewhat bitter-tasting chestnut honey proved to be superior in terms of antibacterial activity in comparison to other honey types from Portugal [16], Spain [17,18], and Turkey [19]. Previous tests by our research group also confirmed that chestnut honey had the highest antibacterial effect, compared to a set of varietal honeys harvested in Hungary, including lavender, linden, acacia, sunflower, milkweed, and goldenrod honeys [20].

Our study aimed to prove the antibiofilm effect of chestnut honey against various combinations of mixed biofilms, using *in vitro* microbiological methods. Our experiments were designed to reveal:

(1) From the combinations *P. aeruginosa*-MRSA, *P. aeruginosa*-*S. epidermidis*, and *S. epidermidis*-MRSA, which were the most sensitive to treatment with chestnut honey;
(2) In which stage of biofilm formation could this honey type interfere the most.

## 2. Results

### 2.1. Melissopalynological Analysis

Based on sensory characteristics and the results of the pollen analysis (Table 1), our honey sample can be considered a variety honey. The color of the honey was amber, its consistency was medium dense, and the taste was bitter. From the pollen spectrum, it can be seen that chestnut (*Castanea*) pollen was present as the dominant pollen, while linden (*Tilia*), sunflower (*Helianthus*), acacia (*Robinia*), and rape (*Brassica*) pollen were also observed in the sample (Table 1). Since *Castanea* pollen is strongly over-represented in chestnut honey, many European laboratories require a percentage of 90% to accept the honey as unifloral [21]. The chestnut pollen percentage calculated for our sample approaches this value, and together with the presence of sensory traits typical for chestnut honey, our honey sample can be accepted as a unifloral honey.

### 2.2. Minimum Inhibitory Concentration (MIC)

During our tests, we observed that the most resistant pathogen was *P. aeruginosa*, where the value of the minimum inhibitory concentration (MIC) was 6%. *Staphylococcus* strains (MRSA, *S. epidermidis*) reacted more sensitively, and an MIC value of 4% was measured for both. Based on the MIC results, during the antibiofilm assay, the combined biofilms of *P. aeruginosa*-MRSA and *P. aeruginosa*-*S. epidermidis* were treated with a 6% honey solution. For the *S. epidermidis*-MRSA pairing, a concentration of 4% was used.

### 2.3. Antibiofilm Effect

**Figure 1:**
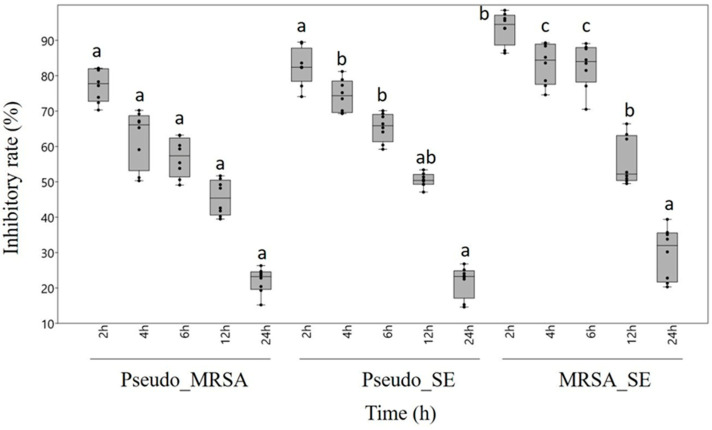
Antibiofilm effect of chestnut honey in the case of combined biofilms of different maturities. Data are expressed using box and jitter plots, minimum to maximum values are presented by vertical lines, while median within the plot as a horizontal line. Jitters represent the data of eight replicates in each box. Lowercase letters a, b, and c above the boxes indicate significant differences between the means of inhibitory rates following incubation for 2, 4, 6, 12, or 24 h, respectively, according to Student’s *t*-test (*p* < 0.01). The same lowercase letters above the boxes indicate mean values that are not significantly different from each other; different lowercase letters indicate means that are significantly different. Pseudo: *Pseudomonas aeruginosa*, MRSA: Methicillin-resistant *Staphylococcus aureus*, SE: *S. epidermidis*.

Regarding the degradation of mixed biofilms, chestnut honey proved to be effective against all three combinations of biofilm-forming pathogens. However, the different maturity statuses of the biofilm influenced the effect exerted by chestnut honey. Based on our results, the more complex the biofilm, the less effective the treatment with honey solution. Following 2 h incubation, biofilm formation was inhibited by 70.3–97.4%, whereas after 4 or 6 h incubation, the rate of biofilm inhibition decreased to 51.2–89%, depending on the combination of treated bacteria. After 12 h of incubation, the moderately developed biofilms were degraded by 39.5–63.4%. Biofilm degradation reached its lowest level in the case of fully developed biofilms, which were left to incubate for 24 hours. The most sensitive was the MRSA-*S. epidermidis* combined biofilm, where we could detect 93.5% inhibition after 2 h of incubation. In contrast, the combination of *P. aeruginosa*-MRSA proved to be the most resistant biofilm, where a significantly lower inhibition rate of 77.2% was measured even in the case of the least mature biofilm (Figure 1).

Furthermore, using the MTT staining procedure, we observed that with the maturation of the biofilm, the percentage of viable cells decreased (Table 2), while that of non-living cells increased. The latter play an important role in the structural construction of biofilms.

**Figure 2:**
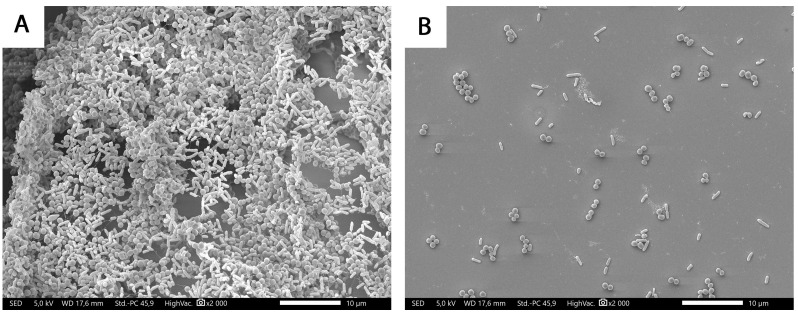
SEM micrographs of *P. aeruginosa*-MRSA mixed biofilm after 6 h of incubation. (**A**) control, (**B**) treated with a 6% chestnut honey solution.

In order to demonstrate the effect of honey treatments at the cellular level, SEM images were taken in the case of the most resistant combined biofilm (*P. aeruginosa*-MRSA). The coherent structure of the developing biofilm (6-h incubation time), in which both MRSA and *P. aeruginosa* take part, is clearly visible in the SEM image of the untreated control (A). As a result of the treatment, the degradation of the biofilm could be observed, and the cells were present separately, in so-called planktonic form (B), which proves the effectiveness of chestnut honey as an antibiofilm agent (Figure 2).

## 3. Discussion

The European Society of Clinical Microbiology and Infectious Diseases (ESCMID) 2014 biofilm diagnostic and treatment guidelines stated that there is a need for new combinations of antibiotics with “biofilm-dissolving” drugs, as well as the search for additional antibacterial and antibiofilm agents [22]. The previously mentioned mixed infections—where more than one strain of bacteria is present—can further complicate the situation. There are many examples of increased antibiotic resistance in mixed-species bacterial biofilms compared to single-species biofilms. For example, in an *in vivo* polymicrobial wound model, *P. aeruginosa* growing in a single-species biofilm was twice as sensitive to gentamicin treatment as *P. aeruginosa* in a polymicrobial biofilm with *S. aureus*, *Enterococcus faecalis*, and *Finegoldia magna* [23]. By physically disrupting the wound biofilm *in vivo*, Wolcott et al. [24] identified a therapeutic window of 24–48 h during which antibiotic therapy was more effective. This indicates that strategies designed to physically disrupt the biofilm may promote antibacterial efficacy.

Our results suggest that treatment with chestnut honey can achieve the disruption of mixed biofilms. Application of honey, preceding or parallel to antibiotic therapy, could be a useful strategy in the treatment of chronic wounds. Honey has been valued since ancient times for its wound-healing properties as well as its antibacterial and antiviral effects [25]. Several compounds are involved in the development of the antibacterial effect of honey, the concentrations of which differ in the case of honeys of different botanical origins. The general antibacterial activity of honey is statistically significantly correlated with the hydrogen peroxide and total polyphenol content of honey [26]. The development of the antibacterial effect of honey can be related to the low pH level and the high sugar content (high osmolarity), which are sufficient to inhibit the growth of microbes. Medical-grade honey has an *in vitro* bactericidal effect against antibiotic-resistant bacteria. The wound-healing effect of honey is also based on the fact that it maintains a moist wound state, and its high viscosity provides a protective barrier to prevent infection. It has a broad-spectrum antibacterial effect, and many of its components can work synergistically, preventing the formation of biofilm and reducing the production of virulence factors [25].

Chestnut honey has outstanding antibacterial and wound-healing properties due to its high content of kynurenic acid, phenolic compounds, hydroxymethylfurfural, and proline, in addition to its relatively high moisture content [27,28,29]. Our previous research results highlighted that chestnut honey was effective against biofilms formed by wound-associated pathogens through membrane degradation and quorum sensing inhibition [30,31]. However, studies so far have not focused on the effectiveness of chestnut honey against mixed biofilms. In the case of wound infections, a biofilm created by more than one strain is usually observed on the surface of the wounds. Most often, *P. aeruginosa*, MRSA, and *S. epidermidis* together create a biofilm on the surface of wounds, which greatly complicates effective treatment with antibiotics. Based on this fact, we created mixed biofilms during our research, which were treated with a chestnut honey solution of a specific concentration.

Previous studies have proven the antibacterial effect of chestnut honey against both Gram-negative and Gram-positive pathogens. Using the tube dilution method, the minimum inhibitory concentration value of chestnut honey against *Staphylococcus aureus* ATCC6538, *Bacillus cereus* ATCC7064, *Escherichia coli* ATCC11293, and *Pseudomonas aeuroginosa* ATCC27853 was determined. In order to support the antifungal effect of chestnut honey, *Candida krusei* ATCC6258 and *C. parapsilosis* ATCC22019 were included in the study. All bacterial strains were found to be sensitive to treatment with chestnut honey, with *B. cereus* being the most sensitive test bacterium. It was found that chestnut honey is more effective against bacteria compared to fungi [19]. Another study tested the effectiveness of avocado, chestnut, and mixed flower honey against *S. aureus* and *E. coli* bacteria. The series of *in vitro* microbiological tests confirmed that the 5% and 10% solutions of all three honey samples were effective after 30 min during the cell viability experiment. In the study, chestnut honey and mixed flower honey proved to be more effective compared to avocado honey. During the membrane potential test, it was proven that avocado honey had a negligible effect, but chestnut honey proved to be promising [18]. Similar to the previous ones, Oliveira’s work group included these pathogens in their experiments, supplemented with the bacterium *P. aeruginosa*. The appearance of chronic wounds was modeled on pig skin. During the study, the synergistic effect of chestnut honey and bacteriophages against mixed biofilms was tested. Their results supported the effectiveness of the combined treatment, which was significant in the case of *P. aeruginosa* [16]. In the treatment of wound infections, in addition to plasters and bandages, the application of different creams is a promising option. Some studies support the effectiveness of gels, creams, and ointments containing chestnut honey. One possible way to treat chronic wounds caused by diabetes is carboxyethyl cellulose hydrogel, which contains chestnut honey (5, 10, 15, 20%). The agar-well diffusion method was used with the inclusion of *S. aureus* and *E. coli* bacteria. During the *in vitro* study, the carboxymethyl cellulose paste containing chestnut honey was tested (CMC-CH). Their results showed that *S. aureus* reacted more sensitively to the treatment, as larger inhibition zones were detected (3.0 mm) than in the case of *E. coli* (2.0 mm). After that, the effectiveness of CMC-CH obtained during the microbiology test was also supported by an *in vivo* mouse experiment [32]. A recent Latvian study compared the effectiveness of different unifloral honey samples. To support the antibacterial effect, an agar-well diffusion test was used. Among the bacteria included in the test were *P. aeruginosa*, *S. aureus*, and MRSA. The antibiofilm effect test was carried out on 96-cell microtiter plates. In terms of the biofilm inhibitory effect, buckwheat honey was followed by chestnut honey when 4 and 24 h of incubation were used, thus ahead of linden, manuka, and thyme honey samples. However, after 48 h of incubation, the efficiency of chestnut honey decreased significantly [33]. Overall, the antibacterial and antifungal effects of chestnut honey should also be highlighted based on previous studies. Moreover, it is important to mention that the use of chestnut honey in the field of wound treatment is one of the most promising alternative complementary therapies. During our study, different mixed biofilms were treated, which, in cases of wound infection, very often colonize the given wound surface, forming a biofilm, thus making treatment difficult. During our tests, different maturity levels of *P. aeruginosa*-MRSA, *P. aeruginosa*-*S. epidermidis*, and MRSA-*S. epidermidis* biofilms were treated using chestnut honey. We observed that the more mature the biofilm, the lower the antibiofilm effect that was detected. As biofilms mature over time, they undergo structural and metabolic changes that can make them more resistant to antimicrobial agents. More mature biofilms may have a more complex and denser matrix, which may limit the penetration of antimicrobial compounds such as those found in chestnut honey. The more mature the biofilm, the more reduced is the metabolic activity of the EPS-encapsulated bacteria. This increases tolerance to antimicrobial agents, as many classes of antibiotics are only effective against actively dividing cells, against peptidoglycan production in the cell wall (β-lactams), protein (aminoglycoside) synthesis, or DNA replication (quinolones) [34]. In addition, the longer the bacterial biofilm develops, the greater the chance of the transfer of antimicrobial resistance genes carried on plasmids [35]. We also concluded that the sensitivity of the biofilm to treatment varies depending on the composition of the biofilm. *P. aeruginosa*-MRSA proved to be the most resistant combination. Biofilms formed by *S. epidermidis*-MRSA bacteria were most effectively suppressed by the chestnut honey treatment. Our results are in line with previous research, as the outstanding antibacterial and antibiofilm effects of chestnut honey have been confirmed.

The novelty of our work is that we succeeded in proving the effectiveness of chestnut honey through the modeling of mixed biofilms and also compared the resistance of biofilms of different degrees of maturity to chestnut honey treatment. We illustrated our results using SEM images. Overall, it can be concluded that our study highlighted the possibility of using chestnut honey to complement antibiotic therapy, as its ability to disrupt the mixed bacterial biofilm of wound-associated pathogens was modeled for the first time.

## 4. Materials and Methods

### 4.1. Honey Sample and Melissopalynological Analysis

The chestnut honey used in our study was obtained from a Hungarian beekeeper (Zala county, Hungary, 2023). The botanical origin of chestnut honey was confirmed by melissopalynological analysis, performed in the same manner as described in Balázs et al. (2023) [20]. 10 g of honey and 20 mL of distilled water was added to a 50 mL centrifuge tube, vortexed, and then centrifuged at 3000 rpm for 10 min (Neofuge 15R centrifuge, Lab-Ex Ltd., Budapest, Hungary). The supernatant was poured off, and then the tube was filled to the mark with distilled water. Following centrifugation again at 3000 rpm for 5 min, the supernatant was poured off and the remaining liquid was drained onto absorbent paper. Microscopic slides were labeled, and a frame corresponding to the size of the cover plate was drawn with a felt-tip pen, and it was preheated for 55 °C on a hotplate (OTS 40, Tiba Kft., Győr, Hungary). To the sediment left in the centrifuge tube, 250 µL of distilled water was added and then vortexed. 50 µL of this suspension was brought to the designated area of the slide with a pipette and spread, then left to evaporate the water from it. After drying, fuchsine-glycerin-gelatin dye was placed on top of the preparation, and after melting, it was covered with a cover plate. The preparations were examined with a Nikon Eclipse E200 microscope (Auro-Science Consulting Kft., Budapest, Hungary) at 400× magnification. Quantitative evaluation was performed by counting at least 500 pollen grains, identifying their source plant at species, genus, or at least family level. As a reference tool, the Bee Pollen Atlas 1.0 was used, which was developed specifically for Hungarian honey samples. Afterwards, the percentage of pollen types was calculated.

### 4.2. Microbiological Assays

#### 4.2.1. Bacterial Strains

The antibacterial effect of chestnut honey was determined on *Pseudomonas aeruginosa* ATCC 27853, *Staphylococcus epidermidis* ATCC 12228, and methicillin-resistant *Staphylococcus aureus* ATCC 700698. Test bacteria were grown in 100 mL sterile BHI (Brain Heart Infusion, Sigma Aldrich Ltd., Budapest, Hungary). Each bacterium was incubated in a shaker incubator (C25 Incubator Shaker, New Brunswick Scientific, Edison, NJ, USA) at 37 °C and at a speed of 60 rpm for 12 h [36]. The bacterial suspensions were diluted with clear BHI to the appropriate concentrations for each assay.

#### 4.2.2. Determination of Minimum Inhibitory Concentration

The antibacterial effect of the chestnut honey sample against each bacterial strain was determined on a 96-well microtiter plate using the microdilution method [37]. The minimum inhibitory concentration (MIC) is the lowest of the test substance concentration that still inhibits the growth of the given bacterial strain. The lower the MIC value, the more effective the tested substance is. Bacterial suspensions (100 µL) of 10^5^ cfu/mL were treated with 100 µL of aqueous honey solutions with different concentrations (stock solutions: 8, 10, 12, 14, 16, 18, and 20%), followed by a 24-h incubation at 37 °C. A cell-free nutrient solution was used as a negative control, the untreated bacterial suspension as a positive control. After completion of the incubation period, absorbance was measured at 600 nm using a microtiter plate reader (BMG Labtech SPECTROstar Nano, Budapest, Hungary). The absorbance value of the treated samples was compared to the absorbance value of the positive (untreated) control. The value where a 90% reduction of bacterial cells was detected was considered the MIC value. The MIC was determined in 8 repetitions.

#### 4.2.3. Antibiofilm Activity

In the first step, the biofilms were formed in a 96-well microtiter plate (10^8^ cfu/mL). In order to model mixed biofilms, 100 µL each of the different bacterial suspensions was added to each well of the microtiter plate. During the research, *P. aeruginosa*-MRSA, *P. aeruginosa*-*S. epidermidis*, and MRSA-*S. epidermidis* combinations were applied. In order to examine the effectiveness of honey against biofilms of different degrees of maturity, different incubation times were used (2, 4, 6, 12, and 24 h). Bacterial cells incubated for 2, 4, and 6 h, were in the initial stage of biofilm formation. Colonies following 12 and 24 h incubation were considered to have moderately and fully developed biofilms, respectively. During the incubation period, the bacterial cells adhered to the walls of the microtiter plate units, forming a biofilm. Bacterial biofilms were washed with physiological saline, then subjected to treatment with 200 µL honey solutions at 6% and 4% concentrations in combinations with *P. aeruginosa* and *S. epidermidis*-MRSA, respectively. After 24 h of incubation, non-adherent cells were washed out, while adherent cells were fixed with 99% methanol, and then biofilms were stained with 0.1% crystal violet dye. After that, the cells were dissolved in 33% acetic acid, and absorbance was measured at 595 nm using a microtiter scanner (BMG Labtech SPECTROstar Nano). Crystal violet is associated with biofilms, binding to negatively charged surface molecules within its extracellular matrix and EPS, thus enabling the measurement of the total biomass of the biofilm in the cell of the microtiter plate. We performed our tests with 6 parallel measurements; the untreated biofilm served as a positive control. The inhibition rate was calculated using the formula below:

Inhibition rate = (1 − S/C) × 100, where S is sample absorbance and C is control absorbance [38]. The biomass of the biofilm serving as the positive control was considered to be 100%, and the degree of inhibition of each honey type was compared to this.

##### Cell Viability in Biofilm Formation

The percentage of living cells can be determined with the MTT (3-[4,5-dimethylthiazol-2-yl]-2,5-diphenyltetrazolium bromide) test, which is a colorimetric assay for measuring the metabolic activity of cells. It is based on the functioning of the mitochondrial respiratory chain. Mitochondrial dehydrogenases reduce yellow MTT to blue formazan crystals [39,40]. Using the crystal violet and MTT assays, the proportion of live bacteria can be determined relative to the total bacteria in the bacterial biofilm of the untreated (positive) control. From this, the proportion of non-living bacteria forming the biofilm can be further deduced. The MTT test was carried out 6 times.

##### Scanning Electron Microscopy (SEM)

In order to visualize our results, we took SEM images of *P. aeruginosa*-MRSA untreated samples, as well as samples treated with different honey solutions (in the case of the *P. aeruginosa*-MRSA and *P. aeruginosa*-*S. epidermidis* combinations: 6% honey solution, *S. epidermidis*-MRSA: 4% honey solution), based on the description of Balázs et al., 2023 [20].

### 4.3. Statistical Analyses

Statistical analyses were carried out using Microsoft Excel^®^ 2016 MSO (16.0.4266.1001) (Microsoft Corp., Redmond, WA, USA). and the PAST software package version 3.11 [41]. The results were expressed as medians (minimum to maximum) and replicated values as indicated. Pairwise comparisons were performed with Student’s *t*-tests. The *p*-values at 1% (*p* ≤ 0.01) were considered significant.

## 5. Conclusions

Due to the ever-spreading antibiotic resistance crisis, it is increasingly urgent to carry out studies on possible alternative therapies with antimicrobial effects. Honey, as an additional therapeutic option with a wound-healing effect, is being used more and more widely. Our tests proved the antibiofilm potency of chestnut honey against mixed bacterial biofilms of wound-associated bacteria, effectively inhibiting biofilm formation in its initial stages and disrupting already developed biofilms. Our study highlights the promising antibiofilm features of chestnut honey, which could be beneficial in the treatment of chronic wounds.

## Figures and Tables

**Table 1:** Pollen spectrum of chestnut honey.

	Pollen Type—Relative Frequency (%)					
Honey type	*Castanea*	*Tilia*	*Helianthus*	*Robinia*	*Brassica*	Other
chestnut	82.9	6.3	0.6	2.4	6.4	1.4

**Table 2:** Percentage of viable cells as a function of biofilm maturity, based on the MTT test carried out in six parallel measurements. Data are shown as means ± standard deviation.

Type of Biofilm	Incubation Time (h)	Viable Cells (%)
*P. aeruginosa*-MRSA	2	89.1 ± 0.2
	4	79.4 ± 0.5
	6	65.7 ± 0.6
	12	59.2 ± 0.2
	24	26.4 ± 0.1
*P. aeruginosa*-*S.epidermidis*	2	86.6 ± 0.5
	4	75.4 ± 0.4
	6	70.2 ± 0.9
	12	48.6 ± 0.7
	24	25.4 ± 0.6
MRSA-*S. epidermidis*	2	87.4 ± 0.7
	4	74.2 ± 0.8
	6	62.1 ± 0.3
	12	53.9 ± 0.5
	24	34.1 ± 0.4

## Data Availability

The data presented in this study are available on request from the corresponding author.
